# Precision Medicine into Clinical Practice: A Web-Based Tool Enables Real-Time Pharmacogenetic Assessment of Tailored Treatments in Psychiatric Disorders

**DOI:** 10.3390/jpm11090851

**Published:** 2021-08-27

**Authors:** Stefania Zampatti, Carlo Fabrizio, Michele Ragazzo, Giulia Campoli, Valerio Caputo, Claudia Strafella, Clelia Pellicano, Raffaella Cascella, Gianfranco Spalletta, Laura Petrosini, Carlo Caltagirone, Andrea Termine, Emiliano Giardina

**Affiliations:** 1 Genomic Medicine Laboratory UILDM, IRCCS Fondazione Santa Lucia, 00179 Rome, Italy; s.zampatti@hsantalucia.it (S.Z.); carlo.fabrizio217@gmail.com (C.F.); giuliacampoli90@gmail.com (G.C.); claudia.strafella@gmail.com (C.S.); raffaella.cascella@gmail.com (R.C.); andreatermine544@gmail.com (A.T.); 2 Department of Biomedicine and Prevention, Tor Vergata University of Rome, 00133 Rome, Italy; michele.ragazzo@uniroma2.it (M.R.); v.caputo91@gmail.com (V.C.); 3 Laboratory of Neuropsychiatry, Department of Clinical and Behavioral Neurology, IRCCS Santa Lucia Foundation, 00179 Rome, Italy; c.pellicano@hsantalucia.it (C.P.); g.spalletta@hsantalucia.it (G.S.); 4 Department of Biomedical Sciences, Catholic University Our Lady of Good Counsel, 1000 Tirana, Albania; 5 Department of Experimental Neuroscience, IRCCS Fondazione Santa Lucia, 00143 Rome, Italy; laura.petrosini@uniroma1.it; 6 Department of Clinical and Behavioral Neurology, IRCCS Fondazione Santa Lucia, 00179 Rome, Italy; c.caltagirone@hsantalucia.it

**Keywords:** neuroPGx, pharmacogenomic, software, OpenArray

## Abstract

The management of neuropsychiatric disorders involves different pharmacological treatments. In order to perform efficacious drug treatments, the metabolism of CYP genes can help to foresee potential drug–drug interactions. The NeuroPGx software is an open-source web-based tool for genotype/diplotype/phenotype interpretation for neuropharmacogenomic purposes. The software provides information about: (i) the genotypes of evaluated SNPs (single nucleotide polymorphisms); (ii) the main diplotypes in CYP genes and corresponding metabolization phenotypes; (iii) the list of neuropsychiatric drugs with recommended dosage adjustment (according to CPIC and DPWG guidelines); (iv) the list of possible (rare) diplotypes and corresponding metabolization phenotypes. The combined application of NeuroPGx software to the OpenArray technology results in an easy, quick, and highly automated device ready to be used in routine clinical practice.

## 1. Introduction

The advancement in treatment of diseases has allowed a great improvement in life quality and expectancy, with a demographic transition in the age of the population. Usually, the elderly suffers from chronic disorders (i.e., diabetes, hypertension, hypercholesterolemia and depressive disorder) with frequent comorbidities. Clinicians are aware of many potential drug–drug interactions (DDI) that are among the principal causes of adverse drug reactions (ADR). It is estimated that the DDI risk increases with the number of drugs administrated: from 13% in people with two concomitant drugs, to 38% with four concomitant drugs, till 82% with eight or more concomitant drugs [1,2].

The mechanisms of DDI and ADR involve the pharmacokinetic (PK) and pharmacodynamic (PD) pathways that are influenced by individual genomic differences. Many polymorphic variants have been identified to predict the individual response to drugs. A great number of variants have been found in genes coding for cytochromes responsible for drug metabolism and some ADR have been associated with specific HLA-haplotypes (i.e., HLA-B*57:01 in Abacavir ADR) [3]. To support the correct administration of drugs in clinical practice, several pieces of software have been developed so far (i.e., Epocrates, iFacts, Lexi-interact, Medscape, Micromedex) [4]. The genetic typing of polymorphisms associated with a differential cytochrome activity is a laboratory routine. Selected polymorphisms in *CYP3A5*, *CYP2B6*, *CYP2C9*, *CYP2C19*, and *CYP2D6* genes can be genotyped to define alleles and diplotypes for prediction of enzyme activity. An ideal pharmacogenomic panel should investigate the least number of variants for the identification of the greatest number of alleles. Different technologies have been developed to improve the timing and the costs of this typing. In this scenario, genetic laboratories can perform the genetic typing for pharmacogenomic purposes with several different technologies (i.e., Real Time PCR, OpenArray, next-generation sequencing).

To improve the usefulness of the reported association between polymorphisms and drug effectiveness, several international consortia have published pharmacogenomics (PGx) guidelines. These guidelines reported pharmacologic adjustments recommended in patients with increased/reduced enzyme activity. The most widely used guidelines were developed by the Clinical Pharmacogenetics Implementation Consortium (CPIC) [5] and the Dutch Pharmacogenetics Working Group (DPWG) [6]. These guidelines are based on a systematic review of the available literature. In CPIC and DPWG guidelines, haplotype in CYP genes (coding for cytochromes) associated with a major/minor enzyme activity are reported with a specific suggestion for drug administration. From this perspective, the guidelines were developed as useful instruments supporting clinicians in pharmacologic treatment. Unfortunately, the reported recommendations may be dissimilar in different guidelines [7]. Furthermore, sometimes administration suggestions included in drug labels are discordant with what is reported in the guidelines [8].

The treatment of neuropsychiatric disorders often requires multi-drug administration. Furthermore, the high frequency of reduced compliance in neurological patients led to the need for a rapid PGx panel to improve the optimization times of drug treatment.

The vast majority of genetic analyses are performed through DNA sequencing. This approach can be carried out by sequencing a subset of genes specifically included in a custom panel (target resequencing) or by analyzing the entire exome. The cost reduction at the moment allows the routine use of the exome analysis and the standardization of the analyses, avoiding the development and validation of specific panels. However, the amount of data generated makes the interpretation phase challenging and virtual filters have been proposed that limit the interpretation to the genes of interest only. Alongside the sequencing platforms, a useful approach in many contexts is the genotyping, the genetic analysis limited to single specific polymorphisms. There are many massive genotyping platforms on the market capable of analyzing over a million polymorphisms. These approaches have been used successfully for GWAS, but their use in genetic diagnostics is limited. We believe that the genotyping platform could be an excellent tool in some areas of genetic diagnostics such as pharmacogenetics.

**Figure 1:**
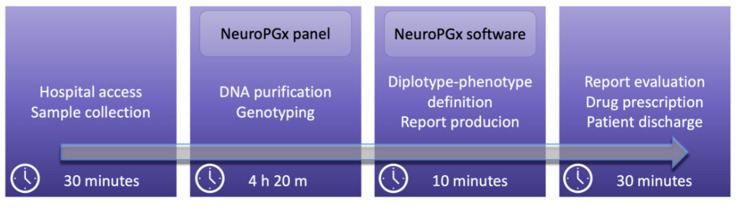
Application model of NeuroPGx system in clinical practice. Excluding the time of admission, clinical visit, and discharge of patients that are highly variable in different clinical centers, the overall time needed by NeuroPGx system is 4 h 30 m.

In this paper, we aimed at describing an integrated NeuroPGx system for the rapid evaluation of samples for neuropsychiatric-pharmacogenomic purposes. The automated genotyping (NeuroPGx panel) is followed by software data analysis and drug administration suggestion (NeuroPGx software), in order to provide useful information in about five hours from the sample collection (Figure 1).

OpenArray™ technology is an easy and quick automated system for high-throughput genotyping. In particular, OpenArray is a nanofluidic real-time PCR system that utilizes a stainless steel, microscopic-scale plate with 384 wells, where TaqMan Assays are spotted according to customer specifications. The plate design allows for the genotyping of several samples in a unique experiment. In fact, the system can process one to four QuantStudio 12K Flex OpenArray plates simultaneously, in order to genotype over 1700 samples in a day.

Similar to what happens in genetic sequencing analyzes, we applied the concept of virtual interpretation panels to genotyping.

The TaqMan^®^ OpenArray^®^ Pharmacogenomics (PGx) Panel analyzes a large number of polymorphisms, but the interpretation is limited only to those of interest for neuropharmacogenomic purposes to reduce the analysis and reporting times. In particular, out of the 60 polymorphisms in 14 genes, 39 SNPs in 5 genes were selected for their known role in the metabolism of neurological drugs. Table 1 summarizes polymorphic variants selected for neuropharmacogenomic (NeuroPGx panel) OpenArray™ panel.

In order to reduce the time for analysis, a user-friendly software for haplotype definition has been created. The NeuroPGx software is an open-source facility for genotype/diplotype/phenotype interpretation for neuroPGx purposes. It has been developed for the OpenArray™ NeuroPGx panel, but it can elaborate data from each genotyping platform.

## 2. Materials and Methods

### 2.1. Selection of Drugs and CYP Variants

Forty-seven neuropsychiatric drugs were selected for their metabolic association with CYP enzymes. One non-neurological drug (clopidogrel) has been added to the list of selected drugs due to its large use in clinical practice. The selected drugs and CYP genes associated with a different drug metabolism are summarized in Table 2.

From all CYP genes, *CYP3A5*, *CYP2B6*, *CYP2C9*, CYP2C19, and *CYP2D6* were selected for their large representation in guidelines. Thirty-nine SNPs were selected in order to define haplotypes associated with differential enzyme activity at *CYP3A5*, *CYP2B6*, *CYP2C9*, *CYP2C19*, and *CYP2D6*. The selected SNPs and allele frequencies across populations [5] are summarized in Table 3.

### 2.2. DNA Purification and Quantification

Genomic DNA was extracted from 400 µL of peripheral blood using MagPurix Blood DNA Extraction Kit and MagPurix Automatic Extraction System (Resnova, Genzano di Roma, Rome, Italy) according to the manufacturer’s instructions. The concentration and quality of the extracted DNA was checked by DeNovix Spectrophotometer (Resnova).

### 2.3. OpenArray™ Technology

The OpenArray™ Technology (Thermo Fisher Scientific, Waltham, MA, USA) is a high-throughput real-time PCR genotyping method that allows a rapid screening of several TaqMan assays in many samples. This real-time method involves the use of an array composed of 3072 through-holes running on QuantStudio 12K Flex Real Time PCR System (Thermo Fisher Scientific) with OpenArray™ block. The OpenArray™ system is composed of a specific plate (OpenArray™ plate) divided into 48 subarrays. The plate customized for this work consists of 60 probes pre-spotted in each of these 48 wells [62].

For each sample, 125–150 ng of extracted DNA and 3μL of 2X TaqMan OpenArray™ Genotyping Master Mix were manually loaded into 384-well plates according to manufacturer’s instructions (Thermo Fisher Scientific). Negative control was obtained by adding 3μL of pure distilled water to the Master Mix. The QuantStudio 12K Flex OpenArray™ AccuFill System transfers the previously generated mix to TaqMan OpenArray™ plate. The amplification is performed by QuantStudio 12K Flex Real Time PCR System (Thermo Fisher Scientific) instrument and the results have been analyzed by the Taqman Genotyper Software v1.3 and verified on Genotype app on Thermo Fisher Cloud (Thermo Fisher Scientific). For each SNP, the call rate was calculated through the Genotype app on Thermo Fisher Cloud.

### 2.4. Statistical Analysis

At the end of the analytical assay, the TaqMan Genotyper Software indicates the percentage of successful genotyping, reported as call rate. The call rate is defined as the fraction of SNPs that were assigned a genotype call by the software compared to the total number of SNPs typed. The successful call rate is defined as more than 90%.

### 2.5. NeuroPGx Software Designing

Based on CPIC allele definition, an automated algorithm (NeuroPGx software) was created for the identification of diplotypes compatible with genotypes at selected SNPs. In particular, a user-friendly system was developed the genotype-diplotype definition for each CYP included in the neuroPGx panel. All possible diplotypes were evaluated for their enzymatic phenotype [9] and the frequency in the reference population [5].

The NeuroPGx software takes subjects’ genotyping on five core genes (*CYP2B6*, *CYP2C19*, *CYP2C9*, *CYP2D6*, *CYP3A5*) as input and calculates all of the possible haplotype combinations, which are more than 5 million. As only a set of diplotypes are assignable to each subject based on their genotype, NeuroPGx outputs a table containing the possible diplotypes and highlights the most probable one based on population frequencies. Moreover, it reports genetic variation-associated drug metabolism profiles (Normal Metabolizer, Poor Metabolizer, Intermediate Metabolizer, Rapid Metabolizer, Ultrarapid Metabolizer). Finally, this information highlights CPIC and DWPG guidelines about drug use for the associated metabolism profiles [5,6,7].

### 2.6. NeuroPGx Software Application

NeuroPGx is written in R programming language (version 4.0.5) and the published interactive app is a dashboard made with Shiny (version 1.6.0) [63,64]. The application can be run locally or on a server. Running NeuroPGx requires installing the latest version of R and Rstudio [64]. NeuroPGx is freely available on GitHub (https://github.com/Andreater/NeuroPGx, accessed on 20 August 2021) and it’s released under the AGPLv3 license.

**Figure 2:**
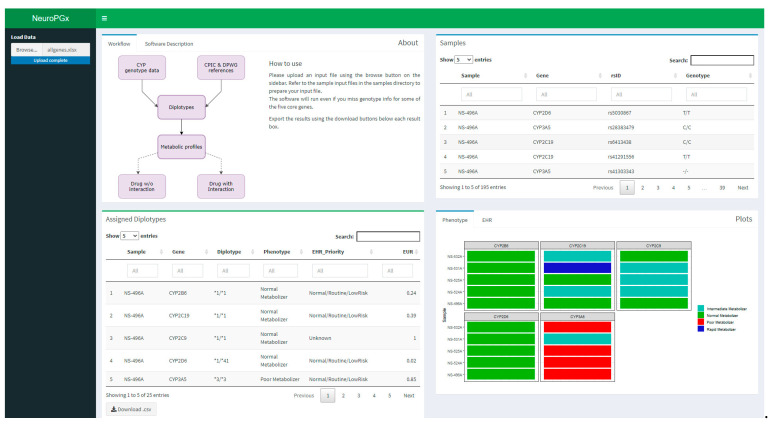
Overview of NeuroPGx software. On the top left, the user control menu. The four panels show: details about how the software works (up left), overview of uploaded samples and genotypes (up right), overview of assigned diplotypes (down left), rapid overview of metabolization profile (down right). Two other panels report guideline suggestions for sample metabolization profile (Figure S2).

Accessing GitHub (https://github.com/Andreater/NeuroPGx, accessed on accessed on 20 August 2021) users can download the software and the instruction files, which are reported on the readme file of the repository. Extensive installation instructions are reported both on the readme and in the package homepage on GitHub. Users can find several examples of genotyping tables in the samples folder of the software (NeuroPGx/data/samples). The genotyping table is the input required to run NeuroPGx and it should contain all genotypes at selected SNPs (Table 3) for one or more samples. The input file can be prepared in an Excel spreadsheet with 4 columns: Sample, Gene, rsID, Genotype. Samples’ ID should be reported in the Sample column while Gene Symbols and dbSNP IDs should fill the Gene and rsID columns. The Genotype column should be filled with genotype information for each sample and a “/” should be used as a separator. Deletions in a SNP can be coded as -/- or A/-, while more complex configurations, such as CTT/CTT, can be easily reported (Table S1). NeuroPGx also accepts .tsv and .csv files. If the genotyping table lacks some SNP or gene, the NeuroPGx will evaluate possible haplotype combinations based on the available data. When the app starts, it needs the genotyping table to be inputted by clicking on the “browse” button at the top left of the page (Figure 2). The elaboration starts and takes about 5-10 min to finish and show the output (Figure S1). The software page is divided into 6 panels. The “How it works” panel contains information about how to use the app. The “Samples” panel reports an interactive view of the input table, while the “Assigned diplotypes” panel shows an interactive view of the output table. The “Plots” panel shows plots for both the phenotype and the EHR, divided into two different tabs. The last two panels, namely “Suggested drug *w*/*o* interaction” and “Suggested drug with interaction” summarize therapy adjustments based on CPIC and DWPG guidelines, as well as literature-retrieved indications. These suggestions are based on subject metabolic profiles without the interaction between genotypes and metabolites or with their interaction, respectively.

## 3. Results

A NeuroPGx system for the rapid pharmacogenomics evaluation of samples was developed and tested. Genotypes of SNPs involved in neuropsychiatric and neurological drug metabolisms (NeuroPGx Panel) were tested on 100 samples through the OpenArray platform, revealing an average call rate of 94.1%. The Taqman Genotyper Software v1.3 on local computer devices and Genotype app on Thermo Fisher Cloud permit the analyzing of genotyping results. Thermo Fisher Cloud is an easy-to-use platform designed to support analyses and interpretation of instrumental results. In particular, Thermo Fisher Cloud gives figurative report of the OpenArray experiments, improving the interpretation and the quality evaluation time. The average call rate and allelic discrimination calls can be visualized in a condensed image that permits the evaluation of the quality of the experiment.

The development and application of NeuroPGx software permits the automated identification of all possible diplotypes compatible with genotypes at each CYP gene included in the virtual NeuroPGx Panel. Based on population characteristics, the software selects the most likely diplotype-phenotype (Table 3 and Figure 2). Otherwise, all possible diplotype-phenotype combinations are identified and listed in the final report. Therefore, the neuroPGx software report provides information about the: (i) genotypes at evaluated SNPs; (ii) main diplotypes at CYP genes and corresponding metabolization phenotypes; (iii) list of neuropsychiatric drugs with recommended dosage adjustment; (iv) list of possible (rare) diplotypes and corresponding metabolization phenotypes.

The application of NeuroPGx software to genotypic data allows one to obtain complete results for a single sample, for a pool of samples, and for samples with incomplete genotype information (i.e., if one gene and/or one SNP is missing). A complete README and “How to use” section on GitHub (https://github.com/Andreater/NeuroPGx, accessed on 20 August 2021) helps users to install and use the software. The software permits the analysis of samples even if the genotypic data are incomplete; detailed examples of how to build the input file (for one sample, for pool of samples, and if one gene/SNP is missing) are included on GitHub (https://github.com/Andreater/NeuroPGx/tree/main/data/samples, accessed on 20 August 2021).

## 4. Discussion and Conclusions

Here, we report the first NeuroPGx system ready for application in clinical practice. The exact interfacing between genotyping technologies and NeuroPGx software allows one to obtain results in a very short time. In particular, the application of virtual NeuroPGx Panel throughout OpenArray™ System to our new NeuroPGx software enables the full analysis (from sample to report) in about 4 h (Figure 1). It is well known that the time of analysis is often crucial in clinical practice and timely therapy of neuropsychiatric disorders can make all the difference. For this reason, the combination of a high-throughput and rapid technology such as OpenArray and the design of an automated interpretation system can improve the effectiveness of pharmacological therapy.

The NeuroPGx panel involves 39 SNPs for cytochrome enzymatic phenotypes’ estimation and prevision of drug response. SNPs were selected from CPIC and DPWG guidelines for pharmacogenomic characterization of patients. These SNPs were tested on 100 samples, revealing an average call rate of 94.1%. The OpenArray technology and the automated interpretation of results through Thermo Fisher Cloud reduce the analysis and interpretation times. In particular, the figurative report of the experiment permits a rapid evaluation of run quality. The evaluation of OpenArray technology confirms that it can easily detect genotypes at multiple loci, allowing one to obtain neuroPGx information in a short time. Compared with classical real-time PCR or with sequencing approaches, the OpenArray technology presents several advantages: it is quite fully automated, it is less time-consuming, and it is easy to analyze. Furthermore, the implementation of data in the Thermo Fisher Cloud facilitates the protection of experimental data from accidental loss. Moreover, the high rate of automated steps in sample processing permits the diffusion of this technology even in laboratories without a high expertise in molecular genetics. Although the processing optimization requires many samples (almost 16), the cost and time are really attractive. In particular, the NeuroPGx panel applied to OpenArray requires less than 4 h for analysis and about 20 min for interpretation.

The NeuroPGx software was designed to provide an automated interpretation of genotyping data that usually require a long time and are subject to human error. The NeuroPGx software is an open-source platform that provides a complete interpretation of genotyping data according to population distribution of compatible CYP alleles. In order to give an accurate and useful interpretation of genotypes, a full evaluation of administration details was performed on neuropsychiatric drugs (Table 2). As well as those of authorities in other countries [8], labels approved by the Food and Drug Administration (FDA), European Medicines Agency (EMA), and Italian Medicines Agency (AIFA) sometimes reported administration suggestions that are discordant with guideline recommendations (Table 4). Thus, the evaluation of cytochrome genotypes and metabolizing phenotypes in clinical practice should take into consideration suggestions of dosage adjustments in order to quickly identify the right dosage for the patient. For this reason, the PGx report provided by NeuroPGx software includes not only genetic data, but also administration recommendations according to the main international guidelines (CPIC and DPWG). Considering the well-known differences among suggestions in guidelines [7], the PGx report highlights all possible recommendations, in order to give complete information useful for clinicians (Figure S2).

To our knowledge, this is the first software that provides complete information from the genotype to the drug administration. Traditionally, interpretation tools help in defining metabolization profiles of selected CYP genes, without any reference to guidelines. The NeuroPGx software is designed to become a useful device in clinical practice; in fact, it is easy to use, it is applicable to data from all genotyping platforms, and it provides updated administration suggestions for all listed drugs.

In conclusion, the combined application of NeuroPGx software to the OpenArray technology results in an easy, quick, and highly automated device ready to be used in clinical practice. In particular, the quickness of this combination will help neurologists and psychiatrists in pharmacological therapy, giving important genetic information in about 4 h. Considering time and costs of the NeuroPGx panel on the OpenArray platform, it is expected that analysis will allow one to obtain useful pharmacogenomic results (with a full genotype-phenotype and drugs report) in less than one working day. Taking into account the high frequency of reduced compliance and the necessity for multi-drug administration in the treatment of neuropsychiatric disorders, a rapid NeuroPGx system will permit the improvement of drug effectiveness. From this perspective, the validation of one-day pharmacogenomic test will promote its diffusion in clinical practice, supporting the precise administration of drugs. Furthermore, the NeuroPGx software by itself can be used to quickly define CYP metabolization profiles and guideline recommendations. Designed to meet the needs of psychiatrists, it will be extended to other pharmaceuticals fulfilling the needs of several specialties.

## Figures and Tables

**Table 1:** NeuroPGx panel (based on OpenArray™ technology).

GENEs	HGVS	Alleles ^1^	Major Nucleotide Variation	RS ID	AssayID	Nucleotide Change	Effect on Protein
*CYP2B6*	NC_000019; NG_007929.1; NM_000767; NP_000758.1	*22, *34, *35, *36	−82T > C	rs34223104	C__27830964_10	g.40991224T > C	Upstream Transcript Variant
		*16, *18	983T > C	rs28399499	C__60732328_20	c.983T > C	p.Ile328Thr
		*5, *7	25505C > T	rs3211371	C__30634242_40	c.1459C > T	p.Arg487Cys
*CYP2C19*	NC_000010; NG_055436; NM_000769; NP_000760	*17	−806C > T	rs12248560	C____469857_10	−806C > T	Upstream Transcript Variant
		*4A/B	1A > G	rs28399504	C__30634136_10	c.1A > G	p.Met1Leu
		*8	12711T > C	rs41291556	C__30634130_30	c.358T > C	p.Trp120Arg
		*6	12748G > A	rs72552267	C__27531918_10	c.395G > A	p.Arg132Gln
		*9	12784G > A	rs17884712	C__25745302_30	c.431G > A	p.Arg144His
		*3	17948G > A	rs4986893	C__27861809_10	c.636G > A	p.Trp212Ter
		*10	19153C > T	rs6413438	C__30634128_10	19153C > T	p.Pro227Leu
		*2	19154G > A	rs4244285	C__25986767_70	c.681G > A/19154G > A	p.Pro227 =
		*7	19294T > A	rs72558186	C__30634127_10	g.94781999T > A	Splice Donor Variant
		*5	90033C > T	rs56337013	C__27861810_10	c.1297C > T	p.Arg433Trp
*CYP2C9*	NC_000010; NG_008385; NM_000771; NP_000762	*2, *35, *61	3608C > T	rs1799853	C__25625805_10	c.430C > T	p.Arg144Cys
		*6	10601delA	rs9332131; hcv32287221	C__32287221_20	c.818delA	p.Lys273ArgfsTer34
		*11	42542C > T	rs28371685	C__30634132_70	c.1003C > T	p.Arg335Trp
		*3, *18	42614A > C	rs1057910	C__27104892_10	c.1075A > G	p.Ile359Val
		*4	42615T > C	rs56165452	C__30634131_20	c.1076T > C	p.Ile359Thr
		*5	42619C > G	rs28371686	C__27859817_40	c.1080C > G	p.Asp360Glu
*CYP2D6*	NC_000022.11; NG_008376; NM_001025161; NP_000097	*10, *36, *37, *47, *49, *52, *54, *57, *64, *65, *69, *72, *87, *94, *95, *99, *100, *101, *114, *132	100C > T	rs1065852	C__11484460_40	c.100C > T	p.Pro34Ser
		*12	124G > A	rs5030862	C__27531552_A0	c.124G > A	P.Gly42Arg
		*17, *40, *58, *64, *82	1022C > T/A	rs28371706	C___2222771_A0	c.320C > T; c.320C > A	p.Thr107Ile; p.Thr107Asn
		*6	1708delT	rs5030655	C__32407243_20	c.454delT	p.Trp152GlyfsTer2
		*8, *14, *114	1759G > A/T	rs5030865	C_30634117D_M0/C_30634117C_K0	c.505G > T;c.505G > C; c.505G > A	p.Gly169Ter; p.Gly169Arg; p.Gly169Arg
		*4	1847G > A	rs3892097	C__27102431_D0	c.506-1G > A	Upstream Transcript Variant
		*3	2550delA	rs35742686	C__32407232_50	c.775delA	p.Arg259GlyfsTer2
		*7	2936A > C	rs5030867	C__32388575_A0	c.971A > C	p.His324Pro
		*32, *41, *69, *91, *119, *123, *132, *138	2989G > A	rs28371725	C__34816116_20	c.985+39G > A	Intron Variant
		*29, *70, *109	3184G > A	rs59421388	C__34816113_20	c.1012G > A	p.Val338Met
		*2, *8, *10, *11, *12, *14, *17, *19, *20, *21, *28, *29, *30, *31, *32, *35, *36, *37, *39, *40, *41, *42, *45, *46, *47, *49, *51, *52, *54, *55, *56, *57, *58, *59, *64, *65, *69, *70, *72, *73, *83, *84, *85, *87, *88, *94, *95, *98, *99, *100, *101, *102, *103, *104, *105, *111, *114, *117, *121, *123, *125, *126, *128, *129, *132, *133, *135, *136, *138	4181G > C	rs1135840	C__27102414_10	c.1457C > G	p.Thr486Ser
		*2, *8, *11, *12, *14, *17, *19, *20, *21, *28, *29, *30, *31, *32, *34, *35, *40, *41, *42, *45, *46, *51, *55, *58, *59, *65, *69, *73, *84, *85, *91, *98, *102, *103, *104, *105, *111, *114, *117, *121, *123, *125, *126, *128, *129, *133, *135, *136, *138	2851C > T	rs16947	C__27102425_10	c.886T > C	p.Cys296Arg
		*9, *109, *115	2616delAAG	rs5030656; HCV32407229	C__32407229_60	c.841_843delAAG	p.Lys281del
*CYP3A5*	NC_000007.14; NG_007938; NM_000777; NP_000768	*8	3699C > T	rs55817950	C__30633872_10	c.82C > T	p.Arg28Cys
		*3	6986A > G	rs776746	C__26201809_30	c.−253-1G > A	Downstream Transcript Variant
		*3	6986A > G + H30Y (3705C > T)	rs776746; rs28383468	C__30633871_50	c.88C > T	p.His30Tyr
		*6	14690G > A	rs10264272	C__30203950_10	c.624G > A	p.Lys208 =
		*9	19386G > A	rs28383479	C__30633863_10	c.1009G > A	p.Ala337Thr
		*7	27131_27132insT	rs41303343	C__32287188_10	c.1035dupT	p.Thr346TyrfsTer3
		*2	27289C > A	rs28365083	C__30633862_10	c.1193C > A	p.Thr398Asn

^1^ according to Pharmacogene Variation Consortium [9].

**Table 2:** Selected drugs and CYP genes associated.

Drug	Genes ^1^	References
Agomelatine	*CYP1A2, CYP2C9*	[10,11]
Alprazolam	*CYP2C19, CYP2C9, CYP3A4, CYP3A5, CYP3A7*	[10,12,13,14]
Amitriptyline	**CYP2D6, CYP2C19, ** *CYP1A2, CYP2C9, CYP3A4*	[10,15]
Aripiprazole	**CYP2D6, ** *CYP3A4*	[10,16,17]
Atomoxetine	**CYP2D6**, *CYP2C19*	[18,19]
Bupropion	*CYP2B6*	[10,15]
Buspirone	*CYP3A4*	[10,20]
Carbamazepine	*CYP1A2, CYP3A4, CYP3A5, CYP2C19, CYP2C8*	[10,21,22,23]
Chlorpromazine	*CYP2D6, CYP2C19, CYP1A2, CYP3A4*	[10,24,25,26]
Citalopram	**CYP2C19, ** *CYP2D6, CYP3A4*	[10,15,27]
Clobazam	**CYP2C19, ** *CYP3A, CYP2B6*	[28]
Clomipramine	**CYP2D6, ** *CYP2C19, CYP1A2, CYP3A4*	[10,15]
Clonazepam	*CYP3A4, CYP3A5*	[10,29]
Clopidogrel	**CYP2C19**	[30]
Clozapine	*CYP1A2, CYP2D6, CYP3A4, CYP2C19*	[10,31]
Desipramine	*CYP1A2, CYP2D6*	[10,32,33]
Desvenlafaxine	*CYP2C19, CYP3A4*	[15]
Doxepin	**CYP2D6, CYP2C19, ** *CYP2C9*	[10,15,34]
Duloxetine	*CYP1A2, CYP2D6*	[10,15,35]
Escitalopram	**CYP2C19, ** *CYP2D6, CYP3A4*	[10,15,36]
Fluoxetine	**CYP2D6, ** *CYP1A2, CYP2B6, CYP2C9, CYP2C19, CYP3A4, CYP3A5*	[10,37]
Fluvoxamine	**CYP2D6, ** *CYP1A2*	[10,15,38]
Haloperidol	**CYP2D6, ** *CYP3A4*	[39]
Imipramine	**CYP2D6, CYP2C19, ** *CYP1A2, CYP3A4*	[10,15]
Lurasidone	*CYP3A4*	[10,15]
Mirtazapine	*CYP1A2, CYP2D6, CYP3A4, CYP2C19*	[10,15,40]
Nortriptyline	**CYP2D6, ** *CYP1A2, CYP2C19, CYP3A4*	[10,33]
Olanzapine	*CYP1A2, CYP2D6*	[10,41]
Oxcarbazepine	*-*	[10,42]
Paroxetine	**CYP2D6, ** *CYP3A4*	[10,15]
Perphenazine	*CYP2D6*	[10,43]
Phenytoin	**CYP2C9, ** *CYP2C19*	[44]
Pimozide	**CYP2D6, ** *CYP3A4, CYP1A2*	[10,45]
Quetiapine	*CYP2D6, CYP3A4*	[10,15,46]
Reboxetine	*CYP3A4*	[10,15]
Risperidone	*CYP2D6*	[10,47,48]
Sertraline	**CYP2C19, ** *CYP2B6, CYP2C9, CYP2D6, CYP3A4*	[10,15,49]
Thioridazine	*CYP2D6*	[10,50]
Trazodone	*CYP2D6, CYP3A4, CYP1A2*	[10,51]
Trimipramine	**CYP2C19, ** *CYP2C9, CYP2D6, CYP3A4*	[10,15,52]
Valproic acid	*CYP2A6, CYP2B6, CYP2C9, CYP2C19*	[10,53]
Venlafaxine	*CYP2C19, CYP2D6, CYP3A4*	[10,15,54]
Vortioxetine	*CYP3A4, CYP2C9, CYP2D6, CYP2C19*	[10,15,55]
Ziprasidone	*CYP3A4*	[10,56,57]
Zolpidem	*CYP1A2, CYP2D6, CYP3A4*	[10,58]
Zonisamide	*CYP3A4, CYP2C19*	[59,60]
Zuclopenthixol	*CYP2D6, CYP3A4*	[10,43,61]

^1^ in bold genes which genotypes suggest a different drug administration, in italic genes associated with a different drug metabolism, but not included in guidelines [10].

**Table 3:** Selected SNPs. Frequency of detectable alleles have been calculated according to population data [5]. Abbr.: SSA = Sub-Saharan African; AA/AC = African American/Afro-Carribbean; Eur = European; NE = Near Eastern; EA = East Asian; CSA = Central/South Asian; Ame = American; Lat = Latino; Oce = Oceanian; AF = allele frequency.

Genes	RS ID	Alleles	SSA AF	AA/AC AF	Eur AF	NE AF	EA AF	CSA AF	Ame AF	Lat AF	Oce AF
***CYP3A5***	rs55817950, rs776746, rs10264272, rs28383479, rs41303343, rs28365083	(*1 *2 *3 *6 *7 *8 *9)	0.54	0.50	0.71	0.62	0.49	0.78	0.83	0.75	0.74
***CYP2B6***	rs34223104, rs28399499, rs3211371	(*1 *5 *7 *16 *18 *22 *34 *35 *36)	0.44	0.52	0.64	0.52	0.66	0.61	n.a.	0.56	0.37
***CYP2C9***	rs1799853, rs9332131, rs28371685, rs1057910, rs56165452, rs28371686	(*1 *2 *3 *4 *5 *6 *11 *18 *35 *61)	86.42	86.70	80.01	76.91	96.57	78.90	88.92	n.a.	96.62
***CYP2C19***	rs12248560, rs28399504, rs41291556, rs72552267, rs17884712, rs4986893, rs6413438, rs4244285, rs72558186, rs56337013	(*1 *2 *3 *4 *5 *6 *7 *8 *9 *10 *17)	0.74	0.75	0.78	0.81	0.98	0.83	0.89	0.82	0.94
***CYP2D6***	rs1065852, rs5030862, rs28371706, rs5030655, rs5030865, rs3892097, rs35742686, rs5030867, rs28371725, rs59421388, rs1135840, rs16947, rs5030656	(*1 *2 *3 *4 *5 *6 *7 *8 *9 *10 *11 *12 *14 *17 *19 *20 *21 *28 *29 *30 *31 *32 *34 *35 *36 *37 *39 *40 *41 *42 *45 *46 *47 *49 *51 *52 *54 *55 *56 *57 *58 *59 *64 *65 *69 *70 *72 *73 *82 *83 *84 *85 *87 *88 *91 *94 *95 *98 *99 *100 *101 *102 *103 *104 *105 *109 *111 *114 *115 *117 *119 *121 *123 *125 *126 *128 *129 *132 *133 *135 *136 *138)	˜1	˜1	˜1	˜1	˜1	˜1	˜1	˜1	0.85

**Table 4:** Comparison between guidelines (CPIC and DPWG) and drug labels (FDA and EMA/AIFA) for pharmacogenetic data. Abbr.: FDA = Food and Drug Administration; EMA = European Medicines Agency; AIFA = Italian Medicines Agency; noRec = no recommendation reported in the guideline; N = no information about CYP phenotypes; Y = the label reports information about CYP phenotypes, with or without suggestion to investigate CYP genotype; NA: not approved.

Drug	Related Genes	Indication in the Guidelines		Label Indication	
		CPIC	DPWG	FDA	EMA/AIFA
Agomelatine		noRec	noRec		
Alprazolam		noRec	noRec		
Amitriptyline	*CYP2D6*			Y	Y
	*CYP2C19*			N	Y
Aripiprazole	*CYP2D6*			Y	Y
Atomoxetine	*CYP2D6*			Y	Y
Bupropion		noRec	noRec		
Buspirone		noRec	noRec		NA
Carbamazepine		noRec	noRec		
Chlorpromazine		noRec	noRec		
Citalopram	*CYP2C19*			Y	Y
Clobazam	*CYP2C19*			Y	Y
Clomipramine	*CYP2D6*			Y	Y
Clonazepam		noRec	noRec		
Clopidogrel	*CYP2C19*			Y	Y
Clozapine		noRec	noRec		
Desipramine		noRec	noRec		NA
Desvenlafaxine		noRec	noRec		NA
Doxepin	*CYP2D6*			Y	NA
	*CYP2C19*			N	NA
Duloxetine		noRec	noRec		
Escitalopram	*CYP2C19*			Y	Y
Fluoxetine	*CYP2D6*			Y	Y
Fluvoxamine	*CYP2D6*			Y	N
Haloperidol	*CYP2D6*			N	N
Imipramine	*CYP2D6*			Y	NA
	*CYP2C19*			N	NA
Lurasidone		noRec	noRec		
Mirtazapine		noRec	noRec		
Nortriptyline	*CYP2D6*			Y	N
Olanzapine		noRec	noRec		
Oxcarbazepine		noRec	noRec		
Paroxetine	*CYP2D6*			Y	Y
Perphenazine		noRec	noRec		
Phenytoin	*CYP2C9*			Y	Y
Pimozide	*CYP2D6*			N	N
Quetiapine		noRec	noRec		
Reboxetine		noRec	noRec		
Risperidone		noRec	noRec		
Sertraline	*CYP2C19*			N	Y
Thioridazine		noRec	noRec		NA
Trazodone		noRec	noRec		
Trimipramine	*CYP2C19*			Y	Y
Valproic acid		noRec	noRec		
Venlafaxine		noRec	noRec		
Vortioxetine		noRec	noRec		NA
Ziprasidone		noRec	noRec		
Zolpidem		noRec	noRec		
Zonisamide		noRec	noRec		
Zuclopenthixol		noRec	noRec		

## Data Availability

The data generated in the present study are included within the manuscript and its supplementary file.

## Supplementary Materials

The following are available online at www.mdpi.com/article/10.3390/jpm11090851/s1, Figure S1: Elaboration images, Figure S2: Full report, Table S1: List of genotypes recognized by the NeuroPgX software.
