# Supplementary material for: Precision Medicine into Clinical Practice: A Web-Based Tool Enables Real-Time Pharmacogenetic Assessment of Tailored Treatments in Psychiatric Disorders
Source: J Pers Med. 2021 Aug 27;11(9):851. doi: 10.3390/jpm11090851 (PMC8471120; doi:10.3390/jpm11090851)
Supplement: Supplementary file 1 [file jpm-11-00851-s001.zip › jpm-1329173-supplementary.pdf]

Figure S1. Elaboration images. Five of the 6 panels of software page show loading image for elaboration time (5-10 minutes)

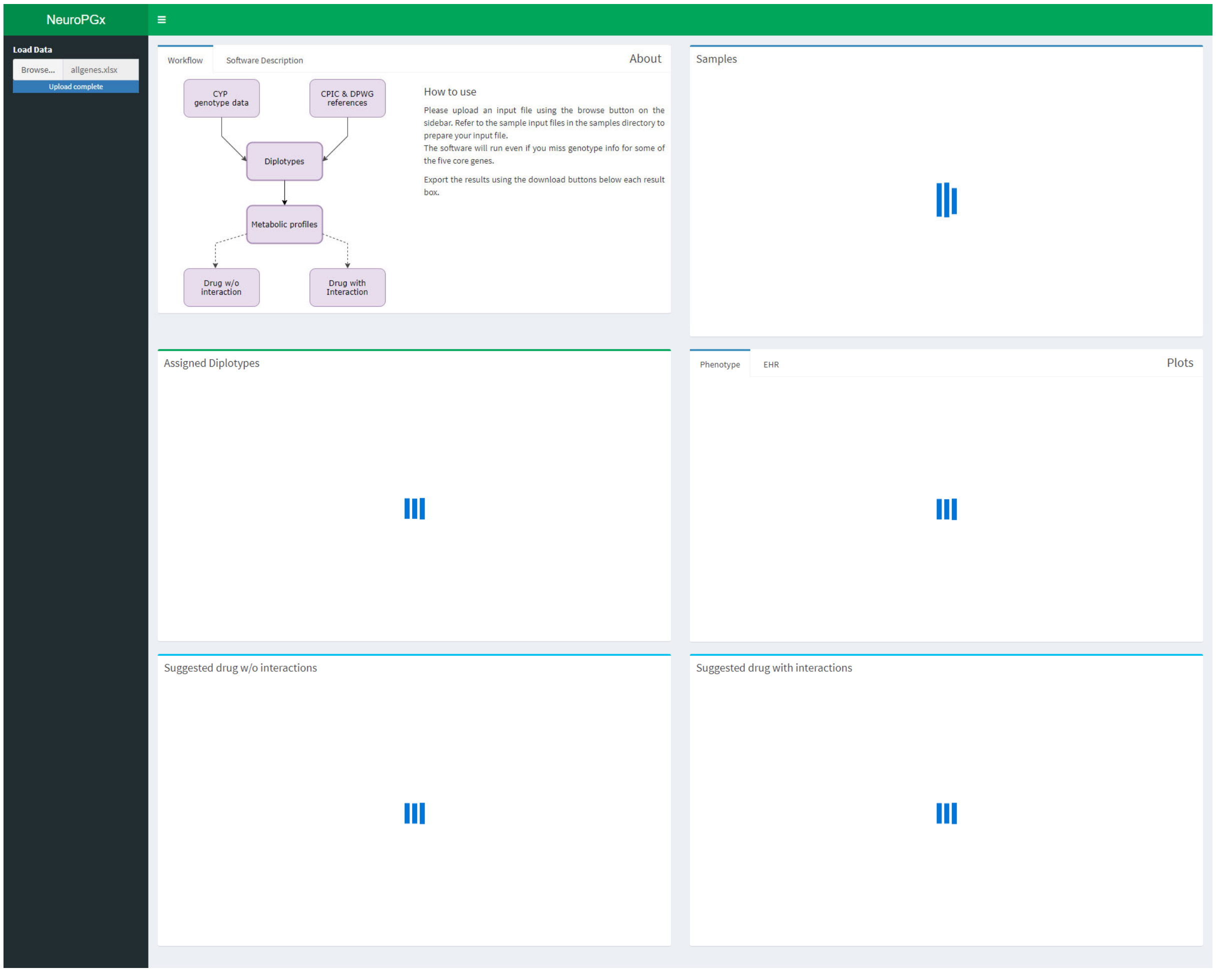

Figure S2. Full report. Overview of the 6 panels after elaboration time. The panels show: (A) details about how the software works, (B) overview of uploaded samples and genotypes, (C) overview of assigned diplotypes, (D) rapid overview of metabolization profile, (E) guideline suggestions for sample metabolization profile (single-gene), (F) guideline suggestions for sample metabolization profile (gene-gene interactions).

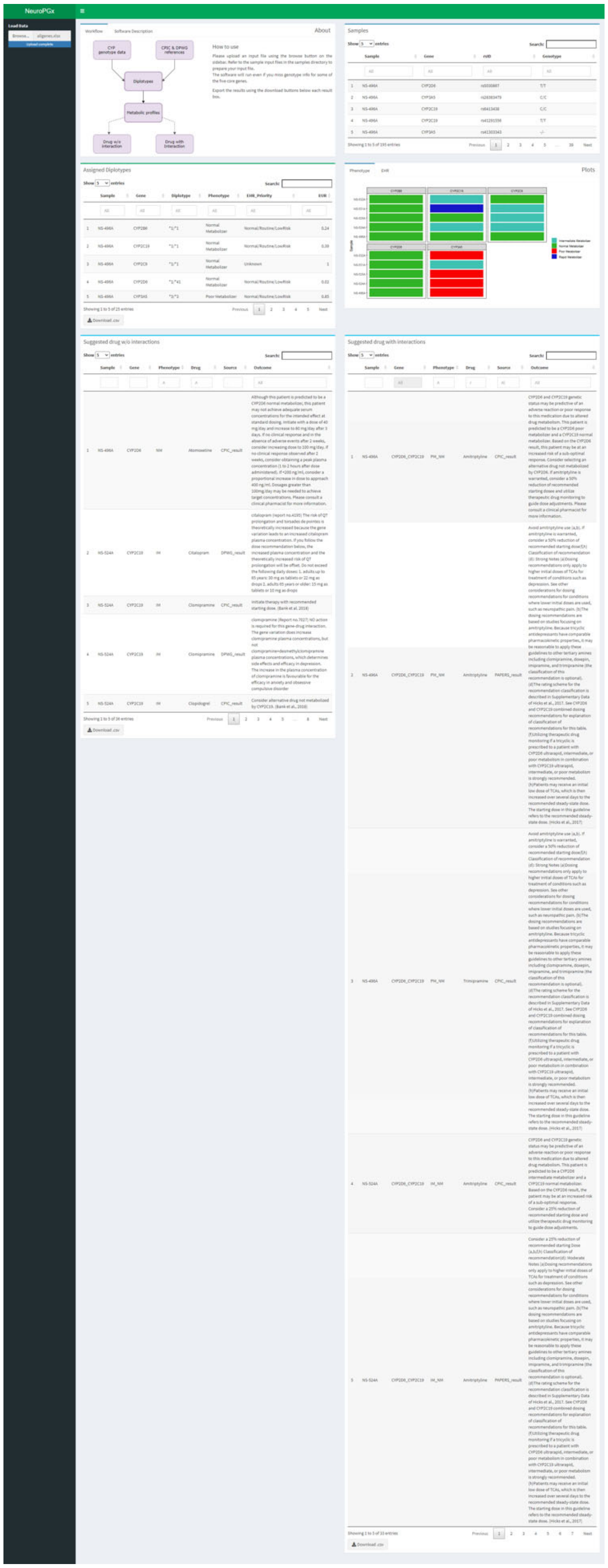

Table S1. List of genotypes recognized by the NeuroPgX software.

CYP2B6	rs34223104	T/T	T/C	C/C			
CYP2B6	rs28399499	T/T	T/C	C/C			
CYP2B6	rs3211371	C/C	T/C	C/C			
CYP2C19	rs6413438	C/C	T/C	T/T			
CYP2C19	rs41291556	T/T	T/C	C/C			
CYP2C19	rs17884712	G/G	A/G	A/A			
CYP2C19	rs72558186	T/T	A/T	A/A			
CYP2C19	rs12248560	C/C	T/C	T/T			
CYP2C19	rs28399504	A/A	A/G	G/G			
CYP2C19	rs72552267	G/G	A/G	A/A			
CYP2C19	rs4986893	G/G	A/G	A/A			
CYP2C19	rs4244285	G/G	A/G	A/A			
CYP2C19	rs56337013	C/C	T/C	T/T			
CYP2C9	rs28371686	C/C	C/G	G/G			
CYP2C9	rs9332131	A/A	A/delA	delA/delA			
CYP2C9	rs1057910	A/A	A/C	C/C			
CYP2C9	rs28371685	C/C	C/T	T/T			
CYP2C9	rs56165452	T/T	C/T	C/C			
CYP2C9	rs1799853	C/C	C/T	T/T			
CYP2D6	rs5030867	T/T	T/G	G/G			
CYP2D6	rs59421388	C/C	C/T	T/T			
CYP2D6	rs28371725	C/C	C/T	T/T			
CYP2D6	rs1065852	G/G	A/G	A/A			
CYP2D6	rs5030655	A/A	A/delA	delA/delA			
CYP2D6	rs5030865	C/C	C/A	A/A	A/T	T/T	C/T
CYP2D6	rs16947	G/G	A/G	A/A			
CYP2D6	rs5030656	CTT/CTT	CTT/delCTT	delCTT/delCTT			
CYP2D6	rs3892097	C/C	C/T	T/T			
CYP2D6	rs28371706	G/G	A/G	A/A			
CYP2D6	rs35742686	T/T	T/delT	delT/delT			
CYP2D6	rs5030862	C/C	C/T	T/T			
CYP2D6	rs1135840	C/C	C/G	G/G			
CYP3A5	rs28383479	C/C	C/T	T/T			
CYP3A5	rs41303343	-/-	insA/-	insA/insA			
CYP3A5	rs776746	C/C	C/T	T/T			
CYP3A5	rs55817950	G/G	A/G	A/A			
CYP3A5	rs28365083	G/G	G/T	T/T			
CYP3A5	rs10264272	C/C	C/T	T/T			
